# Attachment to employment and education before work disability pension due to a mental disorder among young adults

**DOI:** 10.1186/s12888-016-0854-1

**Published:** 2016-05-13

**Authors:** Pauliina Mattila-Holappa, Matti Joensuu, Kirsi Ahola, Jussi Vahtera, Marianna Virtanen

**Affiliations:** Finnish Institute of Occupational Health, Topeliuksenkatu 41 a A, Helsinki, FIN-00250 Finland; Department of Public Health, University of Turku and Turku University Hospital, Turku, Finland

**Keywords:** Mental disorders, Psychiatric diagnosis, Employment, Education, Retrospective, Childhood, Substance use, Comorbidity, Childhood

## Abstract

**Backround:**

We examined attachment to employment and education among young adults before they were granted a fixed-term work disability pension due to psychiatric diagnosis, and the factors associated with this attachment.

**Methods:**

The data comprised all persons aged 18–34 who received a new-onset fixed-term disability pension compensation due to a mental disorder in Finland in 2008 (*N* = 1163). The data were derived from pension applications and the enclosed medical records, and were linked to employment records from a period of three years before the disability pension. We analysed the factors associated with attachment to employment or education with log-binomial regression analysis.

**Results:**

Fifty percent of the participants were attached to employment or education before work disability pension. The attached were more often women; had higher basic and vocational education; had mood disorder rather than psychosis diagnosis as a primary diagnosis; and had no record of harmful alcohol use or drug use, or recorded symptoms of mental disorders already at school-age.

**Conclusions:**

The level of attachment to employment or education before work disability pension is low among young adults with mental disorders and several risk factors predict poor attachment; severe or comorbid mental disorder, early-life psychiatric morbidity, substance use, male sex, low basic education, and lacking vocational education.

## Background

Mental disorders are common during young adulthood and they comprise the majority of causes of work disability pensions in the age group [1–4]. The most common diagnoses associated with work disability pensions among young adults are mood and substance use disorders [4, 5] and psychotic disorders [5, 6]. In Finland, in 2012, mental and substance use disorders comprised 73 % of the disability pensions in the age group of 18–34 years, the most common diagnoses were mood disorders (39 %), schizophrenia and schizotypal and delusional disorders (24 %), and mental retardation (12 %) [7]. These pensions for young people are usually granted as fixed-time pensions, young adults are expected to return to employment or education and rehabilitation during pension should be planned and carried out.

Young adulthood is a period of fundamental change, including developmental tasks of education and starting employment [8–10]. Even though mental disorders in young people can be transient [11] when prolonging, they may hinder the completion of developmental tasks, such as education and attachment to work [12]. Education has been seen as key factor of attachment to employment [13]. Although the labour markets of young adults are uncertain [14, 15] it has been suggested that the majority of young adults are attached to employment, and that those who have not succeeded in attachment to employment are a minority in risk of social exclusion [16]. Studies have shown that unemployment for people with mental disorders is very high [17], and young adults with mental disorders face significant barriers in attachment to employment – as regards both gaining and maintaining employment [18].

The evidence on the risk factors of psychiatric work disability at a young age is sparse. Mental disorders per se have a multifactorial aetiology, involving biological, psychological and social risk factors [10, 19, 20]. Studies among all age groups suggest that work disability has a strong gradient in terms of social inequality, being related to for example low socio-economic status and adverse living conditions, [21–23]. Also psychosocial factors during childhood and adolescence, for example, parental divorce, problems at school, low social support during youth, and alcohol consumption may predispose to psychiatric work disability pension [23, 24]. In all age groups, a longer period of unemployment often preceded psychiatric work disability pension in cases of psychiatric disorders other than depression in Finland [25]. Labour market participation among young adults has been found to decrease with number of childhood negative events, especially for females [26]. However, to date, there is no research that focuses on young adults’ attachment to employment or education before work disability pension due to mental disorders. It is not known whether young adults with long-term work disability have succeeded in gaining work experience before the disability or are they already excluded from the working life. For example, severity of illness might cause difficulties in finding employment and without previous work experience, it might be difficult even with less severe symptoms. This information would be useful in planning the timing and quality of support.

Vocational rehabilitation, should be integrated to clinical treatment to support return to work [27, 28]. Vocational rehabilitation may include e.g. work trials, courses to support employment, supported employment, or job modification. The previous studies on the effects of vocational rehabilitation have not particularly focused on young people. In case there are large numbers of young adults who are granted work disability pension without preceding experience on working life, it is likely that this group of people should be reached earlier and they should be offered vocational rehabilitation with different approach compared to those who receive temporary disability pension after they have gained working experience.

The aims of the present study are:

1. To examine the prevalence of attachment to employment or education before psychiatric disability pension among young adults.
2. To examine which socioeconomic, clinical and life history factors are related to attachment to employment or education.

## Methods

### Participants and procedure

The study is a part of the Young Minds at Work Study, which examines the factors associated with psychiatric work disability and the predictors of returning to labour market among young adults in Finland. Altogether 1163 persons aged 18–34 received a new-onset fixed-term work disability pension compensation due to a mental disorder from a work pension institute in 2008 in Finland*.* There were 1097 persons who were not on family leave, conscripts, in state supported work or self-employed), i.e. they formed the group of people with potential to be attached to paid employment or education. To receive a pension from pension institute, a person has to have worked at least one day in paid employment before receiving the pension. Those who have never been employed receive their benefit from the Social Security Institute of Finland and were not included in the data. The data included persons with ICD-codes F10-F59, F60-F69 and F80-F99 as their primary diagnosis. Cases with diagnoses of F00-F09 (organic mental disorders) and F70-F79 (mental retardation) were excluded.

**Fig. 1:**
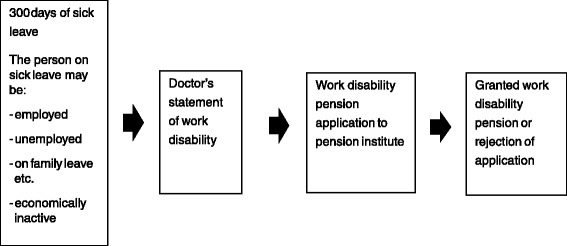
Process of applying work disability pension

To be entitled to apply for work disability pension, the person has to have been disabled for at least 300 days, which is the period of sick leave compensation after which it is possible to apply for work disability pension. The persons applying for work disability pension may be employed, unemployed, or economically inactive (= not seeking for a job), or for example on family leave. The only prerequisite for applying work disability pension is the 300 days of sick leave before applying the pension. This is applicable also to persons without job. The process of disability pension is presented in Fig. 1. Based on personal identification codes the data were derived from the institutes granting pensions (20 work pension institutes or funds). Three researchers collected the data including pension applications and physician-certified medical certificates with their attachments from the pension institutes between September 2012 and June 2013 using a structured electronic Excel sheet which was later transformed into a quantitative dataset. The study was approved by the Ethical Committee of the Hospital District of Helsinki and Uusimaa. The Finnish Ministry of Health and Social Affairs and all the work pension institutes granted permission for the data collection. Two researchers coded 40 cases as duplicates in order to assess inter-rater reliability. The mean agreement for variables used in this study between the two researchers was 92 %.

### Measures

We collected information on socio-demographic, clinical and the life history factors from medical records and work disability pension applications.

Socioeconomic factors: Sex, age (18–24, 25–29, 30–34), basic education (comprehensive school, high school), vocational education (no vocational education, vocational course or apprenticeship, vocational school, university of applied sciences, university).

Clinical factors: Primary diagnosis according to the ICD-10 (International classification of diseases, tenth revision) classification which was further categorised as psychotic, depressive, bipolar, or other mental disorder. The most common diagnoses in the ‘other mental disorder’ group were neurotic, stress-related and somatoform disorders (*N* = 76/137). Psychiatric comorbidity (vs not), somatic comorbidity (vs not), substance use (harmful alcohol use or drug use, vs not), psychiatric hospital admission (at least one vs none), and suicide attempts (at least one vs none).

Life history factors: Symptoms at school age (mentioned in medical records as a contributing factor to the current reason for work disability), mental disorders in family, and childhood adversity (parental divorce, learning difficulties, bullying at school, death or suicide of a parent, parental harmful alcohol use or drug abuse, neglect or sexual abuse, own severe illness, own harmful alcohol use or drug use during childhood, or something else seen as adverse).

Using the person’s identification number, the pension application- and medical records-based data were linked to employment records from the Finnish Centre for Pensions (number of days of employment during the three years preceding the work disability pension). Those with 730 or more days (2 years) of employment during the three years preceding the work disability compensation period were classified as attached to employment. The information on attachment to education was collected from the pension applications. Those enrolled in an educational institute and who stated that this was their main activity at the time of applying for work disability pension were defined as being attached to education.

### Statistical analyses

We calculated first the numbers and percentages of those attached to either employment or education, or solely to employment, and examined the prevalence of attachment according to primary diagnosis. Second, we analysed the factors associated with 1) attachment to employment or education, and 2) attachment to employment, using log-binomial regression analysis by calculating univariate and multivariate prevalence ratios for the studied factors. Those on family leave, conscripts, those in state-supported employment, and self-employed (*n* = 53, 5 %) and those whose current employment or education status was unclear (*n* = 13, 1 %) were excluded from the analysis, since they could not be defined as attached or not attached based on days in employment during three years preceding work disability pension leaving 1097 persons in the study sample. In the first adjusted model, sex and age (as a continuous variable) were added to the log-binomial regression model. In the final adjusted model, we additionally adjusted the models for primary diagnosis class (psychotic, bipolar, depressive, or other mental disorder) and hospital admission as a proxy measure for severity of illness.

The same procedure was replicated with attachment to employment only as the outcome. In this analysis, students (*n* = 229), those who were on family leave or completing military or civil service (*n* = 53) and those whose current employment or education status was unclear (*n* = 13) were excluded, leaving 868 people in the study sample.

## Results

The primary diagnoses (ICD-10 codes) among the study sample are presented in Table 1. Mood disorders (F30-39) were the most frequent diagnosis (54 %), followed by schizophrenia, schizotypal and delusional disorders (34 %), and smaller groups of other disorders.

**Table 1:** Primary diagnosis of young adults with new-onset work disability pension due to mental disorders in 2008 (*N* = 1163)

ICD-10 code	Primary diagnosis	N	%
F10-F19	Disorders due to psychoactive substance use	9	1
F20-F29	Schizophrenia, schizotypal and delusional disorders	400	34
F30-F39	Mood disorders	626	54
F40-F48	Neurotic, stress-related and somatoform disorders	76	7
F50-F59	Behavioural syndromes associated with physiological disturbances and physical factors	25	2
F60-F69	Disorders of adult personality and behaviour	12	1
F80-F89	Disorders of psychological development	8	1
F90-F99	Behavioural and emotional disorders with onset usually occurring in childhood and adolescence and unspecified mental disorder	7	1

### Attachment to employment or education

Descriptive characteristics by socio-demographic, clinical and life history factors and attachment to employment and education before disability pension are presented in Table 2. Of the 1097 people in the study group, 545 (50 %) were attached to either employment or education (Table 2). A total of 362 (33 %) were attached to employment, and 229 (21 %) to education, including 46 people (4 %) who were attached to both employment and education, i.e. they were both enrolled to an educational institute and had been employed at least 730 days during the three years preceding the pension.

**Table 2:** Descriptive characteristics of young adults on work disability pension due to mental disorders according to attachment to employment and education before work disability pension

Characteristics	Attachment to employment or education (*N* = 1097)		Attachment to employment (*N* = 868)	
	n of attached cases/N	% attached	n of attached cases/N	% attached
All	545/1097	49.7	316/868	36.4
Sex				
ᅟMen	207/494	41.9	129/416	31.0
ᅟWomen	338/603	56.1	187/452	41.4
Age (years)				
ᅟ18-24	125/241	51.9	37/153	24.2
ᅟ25-29	189/348	54.3	103/262	39.3
ᅟ30-34	231/508	54.5	176/453	38.9
Basic education				
ᅟComprehensive school	259/634	40.9	183/558	32.8
ᅟHigh school	277/444	62.4	126/293	43.0
Vocational education				
ᅟNo vocational education	204/344	59.3	48/188	25.5
ᅟVocational course or apprenticeship	16/42	38.1	13/39	33.3
ᅟVocational school	177/426	41.5	143/392	36.5
ᅟUniversity of applied sciences	66/107	61.7	57/98	58.2
ᅟUniversity	34/54	64.0	27/47	57.4
Diagnosis				
ᅟPsychotic disorder	142/367	38.7	70/295	23.7
ᅟDepressive disorder	242/433	55.9	151/342	44.2
ᅟBipolar disorder	97/165	58.8	64/132	48.5
ᅟOther mental disorder	64/132	48.5	31/99	31.3
Psychiatric comorbidity				
ᅟYes	269/549	49.0	147/427	34.4
ᅟNo	276/548	50.4	169/441	38.3
Somatic comorbidity				
ᅟYes	43/89	48.3	29/75	38.7
ᅟNo	502/1008	49.8	287/793	36.2
Harmful alcohol use				
ᅟYes	120/304	39.5	74/258	28.7
ᅟNo	425/793	53.6	242/610	39.7
Drug use				
ᅟYes	60/168	35.7	37/145	25.5
ᅟNo	485/929	52.2	279/723	38.6
Psychiatric hospital admission				
ᅟYes	357/720	49.6	199/562	35.4
ᅟNo	188/377	49.9	117/306	38.2
Suicide attempts				
ᅟYes	123/226	54.4	70/173	40.5
ᅟNo	422/871	48.5	246/449	35.4
Symptoms at school-age				
ᅟYes	245/523	46.8	114/392	29.1
ᅟNo	300/574	52.3	202/476	42.4
Mental disorders in family				
ᅟYes	180/336	53.6	115/271	42.4
ᅟNo	365/761	48.0	201/597	33.7
Childhood adversity				
ᅟYes	250/515	48.5	140/405	34.6
ᅟNo	295/582	50.7	176/463	38.0

The unadjusted log-binomial regression models (Table 3) indicated that women were more likely to be attached (PR = 1.34, 95 % CI 1.18-1.52) to employment or education than men. Higher comprehensive education was associated with attachment (PR = 1.53, 95 % CI 1.23-1.72). The prevalence of attachment was higher in the bipolar (PR = 1.52, 95 % Cl 1.27-1.82), depressive (PR 1.44, 95 % CI 1.24-1.68), and other mental disorder groups (PR = 1.25, 95 % CI 1.01-1.56) than in the psychotic disorder group. Absence of harmful alcohol use (PR = 1.36, 95 % CI 1.16-1.58 for non-users) and drug use (PR = 1.46, 95 % CI 1.18-1.81 for non-users) were related to attachment. Age, vocational education, psychiatric or somatic comorbidity, psychiatric hospital admission, symptoms at school-age, suicide attempt, mental disorders in the family, and adverse childhood conditions were not statistically significantly related to attachment in the unadjusted models.

**Table 3:** Associations of socio-demographic, clinical and life history characteristics with attachment to employment or education before work disability pension (*N* = 1097)

Characteristics	PR^a^	(95 % CI)	PR^b^	(95 % CI)^b^	PR^c^	(95 % CI)^c^
Sex						
ᅟMen	1		1		1	
ᅟWomen	1.34	(1.18-1.52)	1.33	(1.18-1.51)	1.23	(1.08-1.40)
Age (years)						
ᅟ18-24	1		1		1	
ᅟ25-29	1.05	(0.90-1.02)	1.04	(0.89-1.21)	1.04	(0.90-1.21)
ᅟ30-34	0.88	(0.75-1.02)	0.88	(0.76-1.03)	0.88	(0.76-1.03)
Basic education						
ᅟComprehensive school	1	1	1		1	
ᅟHigh school	1.53	(1.23-1.72)	1.47	(1.30-1.65)	1.47	(1.30-1.65)
Vocational education						
ᅟNo vocational education	1		1		1	
ᅟVocational course or apprenticeship	0.64	(0.43-0.95)	0.70	(0.47-1.03)	0.69	(0.47-1.02)
ᅟVocational school	0.70	(0.61-0.81)	0.73	(0.63-0.85)	0.73	(0.64-0.85)
ᅟUniversity of applied sciences	1.04	(0.88-1.24)	1.05	(0.88-1.27)	1.05	(0.88-1.25)
ᅟUniversity	1.06	(0.85-1.33)	1.07	(0.86-1.34)	1.02	(0.82-1.28)
Diagnosis						
ᅟPsychotic disorder	1		1		1	
ᅟDepressive disorder	1.44	(1.24-1.68)	1.37	(1.17-1.60)	1.40	(1.19-1.64)
ᅟBipolar disorder	1.52	(1.27-1.82)	1.44	(1.20-1.72)	1.46	(1.21-1.75)
ᅟOther mental disorder	1.25	(1.01-1.56)	1.23	(0.99-1.53)	1.26	(1.01-1.58)
Psychiatric comorbidity						
ᅟYes	1		1		1	
ᅟNo	1.03	(0.91-1.16)	1.04	(0.92-1.17)	1.10	(0.98-1.25)
Somatic comorbidity						
ᅟYes	1		1		1	
ᅟNo	1.03	(0.82-1.29)	1.04	(0.84-1.30)	1.09	(0.88-1.36)
Harmful alcohol use						
ᅟYes	1		1		1	
ᅟNo	1.36	(1.16-1.58)	1.28	(1.09-1.49)	1.32	(1.13-1.54)
Drug use						
ᅟYes	1		1		1	
ᅟNo	1.46	(1.18-1.81)	1.37	(1.10-1.69)	1.33	(1.07-1.64)
Psychiatric hospital admission						
ᅟYes	1		1	1	1	
ᅟNo	1.01	(0.89-1.14)	1.03	(0.91-1.16)	0.94	(0.83-1.07)
Suicide attempts						
ᅟYes	1		1		1	
ᅟNo	0.89	(0.78-1.02)	0.93	(0.81-1.06)	1.00	(0.87-1.14)
Symptoms at school-age						
ᅟYes	1		1		1	
ᅟNo	1.12	(0.99-1.26)	1.18	(1.05-1.33)	1.22	(1.08-1.37)
Mental disorders in family						
ᅟYes	1		1		1	
ᅟNo	0.90	(0.79-1.10)	0.92	(0.82-1.04)	0.95	(0.84-1.07)
Childhood adversity						
ᅟYes	1		1		1	
ᅟNo	1.04	(0.92-1.18)	1.07	(0.95-1.20)	1.09	(0.97-1.20)

PR, prevalence ratio; CI, confidence interval

^a^Unadjusted

^b^Adjusted for age and sex

^**c**^As previous + adjusted for diagnosis and psychiatric hospital admission

In the fully-adjusted (sex, age, diagnosis group, psychiatric hospital admission) model, all the associations remained statistically significant, except for in contrast, symptoms at school-age reached statistical significance (PR = 1.22, 95 % Cl 1.08-1.37) for those with no symptoms) after adjustments.

### Attachment to employment

**Fig. 2:**
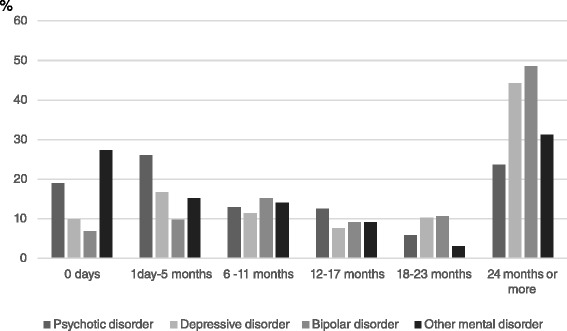
Months of employment during three years preceding work disability pension in different diagnosis groups

Attachment to employment was examined in a group of 868 people, of which 316 (36 %) were attached to employment (had been employed for >730 days) (Table 4), 153 (18 %) had been employed for 365–729 days, 273 (31 %) for 1–364 days, while 126 (15 %) had not been employed at all during the three years preceding the work disability pension. The distribution of days of employment in different diagnosis groups is presented in Fig. 2. The percentage of those without any days of employment during the three years preceding work disability pension was highest among the ‘other mental disorder’ group. Those with mood disorders were most often attached to employment. Based on unadjusted regression models (Table 4), the young adults who were attached to employment before work disability pension were more often women (PR = 1.33, 95 % CI 1.14-1.60), belonged to the oldest age group of 30–34 years (PR 1.61, 95 % Cl 1.19-2.22) and to the second oldest (PR 1.63, 95 % Cl 1.18-2.24) age group rather than to the youngest (18–24), had higher basic (PR = 1.31, 95 % CI 1.10-1.57) and vocational education, with the highest prevalence among those with university of applied sciences (PR = 2.28, 95 % Cl 1.69-3.06) and university (PR = 2.25, 95 % Cl 1.59-3.18) degree (Table 4). The primary diagnosis was associated with attachment, with the highest prevalence being among the bipolar (PR = 2.04, 95 % CI 1.56-2.68) and depressive (PR = 1.86, 95 % CI 1.47-2.36) disorder groups. Absence of harmful alcohol use (PR = 1.38, 95 % CI 1.12-1.72) and drug use (PR = 1.51, 95 % CI 1.13-2.03) were related to attachment. The absence of symptoms at school-age was also related to attachment (PR = 1.46, 95 % Cl 1.21-1.76). Not having a record of mental disorders in the family was associated with a lower prevalence of attachment (PR = 0.79, 95 % Cl 0.66-0.95) compared to those with a record of mental disorders in the family. Psychiatric or somatic comorbidity, psychiatric hospital admission, suicide attempt, and adverse conditions in childhood were not related to attachment to employment.

**Table 4:** Associations of socio-demographic, clinical and life history characteristics with attachment to employment before work disability pension (*N* = 868)

Characteristics	PR^a^	(95 % CI)	PR^b^	(95 % CI)^b^	PR^c^	(95 % CI)^c^
Sex						
ᅟMen	1		1		1	
ᅟWomen	1.33	(1.14-1.60)	1.33	(1.11-1.59)	1.18	(0.99-1.42)
Age (years)						
ᅟ18-24	1		1		1	
ᅟ25-29	1.63	(1.18-2.24)	1.61	(1.17-2.20)	1.60	(1.17-2.19)
ᅟ30-34	1.61	(1.19-2.22)	1.60	(1.18-2.16)	1.56	(1.16-2.10)
Basic education						
ᅟComprehensive school	1		1		1	
ᅟHigh school	1.31	(1.10-1.57)	1.26	(1.06-1.51)	1.29	(1.08-1.53)
Vocational education						
ᅟNo vocational education	1		1		1	
ᅟVocational course or apprenticeship	1.31	(0.79-2.17)	1.27	(0.76-2.11)	1.23	(0.74-2.03)
ᅟVocational school	1.43	(1.08-1.89)	1.39	(1.05-1.83)	1.34	(1.01-1.76)
ᅟUniversity of applied sciences	2.28	(1.69-3.06)	2.07	(1.51-2.84)	1.98	(1.45-2.70)
ᅟUniversity	2.25	(1.59-3.18)	2.08	(1.45-2.97)	1.85	(1.30-2.64)
Diagnosis						
ᅟPsychotic disorder	1		1		1	
ᅟDepressive disorder	1.86	(1.47-2.36)	1.75	(1.37-2.22)	1.80	(1.40-2.30)
ᅟBipolar disorder	2.04	(1.56-2.68)	1.92	(1.46-2.52)	1.95	(1.49-2.57)
ᅟOther mental disorder	1.32	(0.92-1.89)	1.26	(0.86-1.80)	1.31	(0.91-1.89)
Psychiatric comorbidity						
ᅟYes	1		1		1	
ᅟNo	1.11	(0.93-1.33)	1.15	(0.96-1.36)	1.25	(1.05-1.49)
Somatic comorbidity						
ᅟYes	1		1		1	
ᅟNo	0.94	(0.69-1.26)	0.96	(0.71-1.28)	1.06	(0.79-1.41)
Harmful alcohol use						
ᅟYes	1		1		1	
ᅟNo	1.38	(1.12-1.72)	1.30	(1.04-1.62)	1.38	(1.11-1.71)
Drug use						
ᅟYes	1		1		1	
ᅟNo	1.51	(1.13-2.03)	1.39	(1.03-1.87)	1.31	(0.98-1.75)
Psychiatric hospital admission						
ᅟYes	1		1		1	
ᅟNo	1.08	(0.90-1.29)	1.05	(0.88-1.25)	0.92	(0.77-1.11)
Suicide attempts						
ᅟYes	1		1		1	
ᅟNo	0.88	(0.71-1.08)	0.86	(0.70-1.05)	0.97	(0.79-1.19)
Symptoms at school-age						
ᅟYes	1		1		1	
ᅟNo	1.46	(1.21-1.76)	1.41	(1.17-1.71)	1.46	(1.21-1.76)
Mental disorders in family						
ᅟYes	1		1		1	
ᅟNo	0.79	(0.66-0.95)	0.80	(0.67-0.95)	0.85	(0.71-1.01)
Childhood adversity						
ᅟYes	1		1		1	
ᅟNo	1.10	(0.92-1.31)	1.10	(0.92-1.30)	1.14	(0.96-1.35)

PR, prevalence ratio; CI, confidence interval

^a^Unadjusted

^b^Adjusted for age and sex

^c^As previous + adjusted for diagnosis and psychiatric hospital admission

In the fully-adjusted (sex, age, diagnosis group, psychiatric hospital admission) model, the associations remained statistically significant except for those for female sex and mental disorders in the family. After adjustments, not having psychiatric comorbidity was related to attachment to employment. (PR = 1.25, 95 % CI 1.05-1.49).

## Discussion

This study of young adults’ on a fixed-term psychiatric work disability pension showed that 50 % of them were not attached to employment or education before the work disability pension was granted; only a third were attached to employment and every fifth to education. We also found that attachment was associated with different socio-demographic and clinical factors. Having a depressive or bipolar disorder as the primary diagnosis, and no record of substance use or psychiatric symptoms at school-age were associated with a greater likelihood of attachment to employment or education. Furthermore, women were more often attached, as well as those with higher basic and vocational education. Also, absence of psychiatric comorbidity was related to attachment to employment.

To our knowledge, this is the first study on attachment to employment or education before psychiatric work disability pension among young adults. Both attachment to education and employment may be seen as development tasks or parts in a process of attachment to society, as an opposite to exclusion or marginalization. Attachment to employment before work disability pension may be seen as beneficial, since it indicates, that person has, at least partly, succeeded in the developmental tasks of education or employment, and because of positive effects of employment on mental health [29]. In many cases, attachment to education may be highly peer-based since social relations and environment during school years are likely to influence career plans and later on, have an effect on attachment to employment. Planning and executing interventions, especially vocational ones is likely to be easier in case the person already has contact to working life or educational institute since the resources of the work place (e.g. occupational or student health care) are available. Also, the results indicate that many unbeneficial socio-economic and clinical characteristics are associated with not being attached to work before work disability pension.

Primary diagnosis was found to be a fundamental factor relating to attachment; the prevalence of attachment being higher among those with a mood disorder, including participants with bipolar and depressive disorders, than among those with a psychotic disorder. This finding is in line with a previous study, which found that among a population of all age groups, a longer period of unemployment often preceded the work disability pension among those with schizophrenia, than among those with a depressive disorder [25]. As earlier studies have associated bipolar disorders with a high rate of suicide attempts [30–32], comorbidity and disruptive behavioural disorders [32], and cognitive impairment [33], our finding that those with bipolar disorders were most often attached to employment and education compared to the other diagnosis groups is surprising. It seems that those with a bipolar disorder have been able to gain and maintain employment before the onset or worsening of a disorder leading to work disability pension. The percentage of those not being employed at all during the three year period preceding work disability pension was highest among those in the other mental disorder category. The most common diagnoses in this group were neurotic, stress-related or somatoform disorder. This result suggests that these disorders, which include e.g. diagnoses of social phobia, may be associated with severe difficulties to participate in work life long before work disability pension.

Psychiatric comorbidity was related to poor attachment to employment, which suggests that psychiatric comorbidity may cause difficulties gaining and maintaining employment. The prevalence of somatic comorbidity was relatively low in this study group, and was not associated with any form of attachment. An earlier study of a population of all age groups showed that comorbid psychiatric disorder, cardiovascular disease, chronic hypertension, and musculoskeletal disorders were all associated with an increased risk of recurrent sickness absence and fixed-term disability pension episodes [34]. Because research on the contribution of comorbidities in labour market outcomes of young adults is lacking, further studies should analyse this issue in more detail, for example by assessing the severity and type of psychiatric and somatic comorbidity. Harmful alcohol use and drug use were strongly associated with not being attached to employment or education. Previous studies indicate that substance use is connected to both psychiatric and all-cause work disability pension among men under forty [24, 35]. Unlike physical illnesses, mental disorders are characterized by an early onset. Most adults with common mental disorders report their symptoms before the age of 24 [11]. In our study, absence of symptoms at school-age was related to attachment to employment or education, supporting the view that early onset may hinder the possibilities to fulfil the developmental tasks of young adulthood. Furthermore, the disorders with early onset may be more severe than those with later onset [29, 36]

Hospital admission and suicide attempts were not related to attachment to employment or education, nor were recorded mental disorders in the family. Hospital admission may be considered a proxy measure of disorder severity, but it is also likely that hospital treatment has been beneficial for the patients and improved their work ability. However, psychiatric comorbidity and symptoms reported already in childhood – both associated with attachment – may also be seen as measures of severity of illness. It is likely that suicide attempts just preceding work disability pension are associated with not being attached, while those long before might not be. We did not have exact data on the timing of suicide attempts, which should be examined in future studies in more detail.

Recorded adverse childhood factors were not associated with attachment to employment or education in our study. This could be due to the yes/no classification and varying severity of recorded incidents, for example neglect or sexual abuse being more severe than parental divorce. The labour market participation of young adults has been found to decrease with number of negative events [26]. Physical and sexual abuse, and neglect in childhood are linked with common psychiatric disorders in later life [20] but also less severe problems in childhood have been linked with adult depression [37, 38], and parental divorce has been shown to be a risk factor for later work disability [23, 39].

Earlier studies have indicated that disability pensions have a strong socio-economic gradient [21–23]. Our study suggests that socio-economic factors also play an important role in attachment to employment among young adults with a mental disorder. Both higher basic and vocational education were related to a higher likelihood of attachment to employment and education. Furthermore, women were more likely to be attached to employment or education than men. It seems that despite a larger number of women among those who have been granted disability pension in the studied group, women’s situation as regards attachment to employment or education is better than that of men.

This study focused on young adults who received a new-onset fixed-term work disability pension compensation due to a mental disorder from a work pension institute*.* To receive a pension from a work pension institute, the person must have life-time work experience for at least of one day in paid employment before disability pension. Those who had never been employed were not included in our study sample. The strengths of the study were the extensive data, which included 98 % of all the new psychiatric fixed-term work disability pension cases from work pension companies. Inter-rater reliability was also good. The data were based on work ability applications and medical records, the quality of which has been found to vary greatly [40]. However, very detailed information about clinical characteristics and life history was obtained. The days in employment were based on the register of Finnish Centre of Pensions. We did not have information about the content of work or working hours which would also give information about attachment to working life. This study did not have a prospective design, thus causality between examined variables cannot be confirmed.

## Conclusions

In conclusion, fifty percent of young adults with a fixed-term disability pension were attached to employment or education before the work disability pension. The risk factors for poor attachment were psychotic disorder as the primary diagnosis, early onset of symptoms, substance use, and low education, and as regards attachment to employment, also psychiatric comorbidity. This study gives preliminary information for vocational rehabilitation by showing that needed intensity of rehabilitation might be different among those attached and not attached. Young adults who have no connection to education or labour market are a challenge in terms of rehabilitation, because they have risk factors that may predict poor prognosis, such as high psychiatric comorbidity, substance use disorders, and lacking vocational education. They would need intensive interventions which integrate clinical treatment with vocational interventions to support gaining and maintaining employment, while those attached to employment or education may be supported also by lighter interventions executed in co-operation with occupational health care or student health care. Support for families and early interventions in childhood might help prevent later work disability. The strong link between substance use and not being attached to employment or education is of great concern. Evidence-based interventions for comorbid conditions, especially substance use disorders [41] should be part of young adults’ psychiatric treatment when needed and could be beneficial concerning attachment to employment or education. This group of young adults is especially challenging considering participation in working life and needs intensive support in integration to employment, most likely also work opportunities in other settings than competitive employment. Integrating student counselling and career guidance into psychiatric treatment could help in preventing exclusion from employment. Future studies should focus on the reasons behind exclusion from employment already before sick leave and work disability pension, and determine which factors predict return to work after the fixed-term work disability pension among young adults.

### Ethics

The study was approved by the Ethical Committee of the Hospital District of Helsinki and Uusimaa. The Finnish Ministry of Health and Social Affairs and all the work pension institutes granted permission for the data collection.

### Consent to participate

This study was based on register data, thus the participants were not contacted and informed consent was not obtained.

### Consent to publish

Not applicable.

### Availability of data and materials

According to the Finnish Legislation, this type of sensitive and confidential data can only be made available for researchers who have obtained permission from data authorities. For further information, please contact Prof. Marianna Virtanen, Finnish Institute of Occupational Health.

## Abbreviation

ICD-10: international classification of diseases tenth revision
